# The Good, the Bad, and the Rare: Memory for Partners in Social Interactions

**DOI:** 10.1371/journal.pone.0018945

**Published:** 2011-04-29

**Authors:** Jenny Volstorf, Jörg Rieskamp, Jeffrey R. Stevens

**Affiliations:** 1 Max Planck Institute for Human Development, Center for Adaptive Behavior and Cognition, Berlin, Germany; 2 Department of Psychology, University of Basel, Basel, Switzerland; University of Zürich, Switzerland

## Abstract

For cooperation to evolve via direct reciprocity, individuals must track their partners' behavior to avoid exploitation. With increasing size of the interaction group, however, memory becomes error prone. To decrease memory effort, people could categorize partners into types, distinguishing cooperators and cheaters. We explored two ways in which people might preferentially track one partner type: remember cheaters or remember the rare type in the population. We assigned participants to one of three interaction groups which differed in the proportion of computer partners' types (defectors rare, equal proportion, or cooperators rare). We extended research on both hypotheses in two ways. First, participants experienced their partners repeatedly by interacting in Prisoner's Dilemma games. Second, we tested categorization of partners as cooperators or defectors in memory tests after a short and long retention interval (10 min and 1 week). Participants remembered rare partner types better than they remembered common ones at both retention intervals. We propose that the flexibility of responding to the environment suggests an ecologically rational memory strategy in social interactions.

## Introduction

Which do you remember better, an interaction partner who treated you nicely or one who harmed you? Here, we investigated which kind of partner type people remember preferentially, the “good” or the “bad”.

Humans cooperate in a variety of contexts (e.g., [1]), although cooperators risk exploitation by cheaters' accepting but not repaying the beneficial act. One mechanism proposed to explain cooperation between genetically unrelated individuals is reciprocal dependence in repeated interactions: For a cheater who will meet the exploited partner again, the costs of future withheld cooperation by that partner may outweigh the benefits of the current exploitation [2]–[3]. A prerequisite for this direct reciprocity is to identify each partner and remember the history of interactions—an error-prone memory would invite partners to cheat.

Although memory of the partners' behavior is an important building block for the emergence of cooperation [4], storing all actions of all partners is not feasible for the boundedly rational human mind [5]. Time (i.e., the delay until the next access to the information in memory) causes information traces to decay [6], and new and existing knowledge interferes with accurate recall. A study by Stevens, Volstorf, Schooler, and Rieskamp [7] showed that even tracking the single last action of each interaction partner, as many of the proposed reciprocal strategies such as Tit-For-Tat demand [8], led to high memory error rates. In an evolutionary simulation, these errors resulted in a sharp reduction in cooperation. So, remembering either the complete interaction history or the single last action of each partner seem to be ruled out as potential candidates for the memory processes underlying cooperation. An alternative strategy could be to categorize partners into types reflecting their general behavior, for example “cooperator” and “defector”, and remember these types. Compared to constantly updating each partners' actions, the type is a more stable criterion and, therefore, decreases memory effort. Although categorizing partner types may ease memory requirements, the information on partner types is susceptible to forgetting, too. Here, we investigated two hypotheses, the “cheater-memory” and the “rarity” hypothesis, that both propose to remember one partner type preferentially and infer the other, thereby reducing memory load. Barclay [9] and Bell, Buchner, and Musch [10] also addressed these hypotheses, and we extended their approaches by giving participants repeated experience with their partners and testing memory after both a short and long retention interval.

### 1.1 Remember cheaters

One hypothesis predicts that, to reduce fitness costs associated with exploitation, individuals will remember cheaters preferentially. According to error management theory [11], exploitation by a defector is worse than missing out on a cooperative opportunity. To prevent exploitation, individuals would not only benefit from detecting cheaters [12], but because more important information has priority in memory than less important one [13], they would also benefit from remembering cheaters preferentially [14]. We term this the cheater-memory strategy.

In an environment with a majority of cooperators, preferentially remembering the few cheaters reduces the probability of memory errors and related costs. Some environments, however, may contain a majority of cheaters and here, adhering to the cheater-memory strategy would not reduce memory load much.

### 1.2 Remember the rare type

The second hypothesis emphasizes the costs of memory rather than the costs of exploitation. Individuals cope with a variety of environments and, so, rather than always remember cheaters, they might benefit from a memory strategy that adapts to different contexts. Such an ecologically rational [15] strategy would preferentially remember the less frequent partner type [9]. This does not just reduce the amount of information to retain but also potential memory errors. We term this the rarity strategy.

In addition to reducing memory load, focusing on the rare type could be beneficial, because it is novel and striking. Since 1933 [16], researchers have investigated why people better remember distinctive events. The reason, according to Hunt [17], is not an objects' property but the objects' processing via increased attention and memory. Schmidt [18] proposed the incongruity hypothesis, which provides a combination of property- and process-explanations and allows adaption to the environment. Given this definition, one partner type may be preferentially remembered in one context (i.e., an interaction group where it is in the minority) but not in another (i.e., an interaction group where it is in the majority).

### 1.3 Testing cheater-memory and rarity strategies

With this study, we explored two hypotheses regarding which partner type people remember preferentially.

1. According to the cheater-memory strategy, cheaters are remembered better than cooperators, regardless of the number of cheaters or cooperators in the environment.
2. According to the rarity strategy, the rare partner type in an environment is remembered better than the common one; for example, people remember cheaters better only when these are in the minority in the environment.

Adhering to the basic procedure of partner-type memory studies since the seminal paper by Mealey et al. [14], we evaluated participants' memory of the partners by mixing the faces we had presented to participants, the old faces, with new faces. Then, for each face, we asked whether participants had seen it in the beginning of the experiment (*recognition*; e.g., [14], [19]–[20]). Incorporating Mehl and Buchner's [21] suggestion that recognition alone cannot be evolutionarily beneficial, because it does not allow a sufficient partner identification, we additionally asked whether the partner was a defector or cooperator (*categorization*). To test the hypotheses, we varied the proportion of partner types in the interaction group. This has also been done by Barclay [9] and Bell et al. [10] who each found support for a rarity strategy. The design of both studies, however, left open two questions that we addressed here.

#### 1.3.1 Does experience influence categorization?

To indicate that partners are cooperators or defectors, some researchers provided each partner's face with a description of cooperative or noncooperative behavior (e.g., [14], [20], [22]). Barclay and Lalumière [19] criticized these descriptions, as participants could perceive the degree of cheating as higher as that of cooperation, which could lead to a stronger encoding of the cheaters. Moreover, we believe that behavior descriptions likely do not have a large enough impact on participants' behavior and memory (see [23] concerning the importance of the perspective on the cheater-detection mechanism in social-contract violations). In contrast, testing partner-type memory using an economic game (e.g., [24]–[25]) has two advantages. First, games avoid uncertainties about the degree of cooperation and defection. In a Prisoner's Dilemma game, for example, cooperation and defection are not indicated by example descriptions but one of two choices (cooperate/defect) the partner takes, and these choices are associated with a payoff matrix. Second, participants experience the consequences of their partners' behavior directly. Rather than having participants evaluate whether partners with little relation to their welfare have violated social contract or hazard management rules, using a game affects participants immediately, because the payoff depends on their own and the partner's decision. Compared to pure behavior descriptions, the strategic nature of the Prisoner's Dilemma likely triggers behavior and memory processes for tracking cooperators and defectors.

Barclay [9] and Bell et al. [10] employed economic games, but in a limited way. Barclay [9] only announced to participants what their partners would do in a trust game that followed the memory test. Bell et al. [10] emphasized the importance of personal involvement for partner-type memory and let participants experience their partners in a trust game, but it was one-shot and gave participants just a single instance of the type of partner they were facing. We believe it is more realistic if participants are not just confronted with a label or a one-time experience but meet their partners repeatedly [26]. This enables participants to infer the partners' types on their own and increases the recognition accuracy, as people remember self-generated items better than ready-made ones [27]. Repeated interactions also mimic situations outside the lab in which remembering with whom to cooperate and with whom not to cooperate is the prerequisite for establishing reciprocal relationships.

#### 1.3.2 How robust are the memory strategies to longer retention intervals?

The majority of studies on partner-type memory tested recognition (and categorization) in a memory test after either several minutes [9], [20], [22], [25], [28] or 1 week [14], [19], [24] following the initial presentation of the partners by mixing the familiar with new partners. We investigated whether the memory effort associated with longer retention intervals influences the memory strategies. Thus, we asked participants for recognition and categorization of partners after retention intervals of both 10 min and 1 week. Though others have tested the effect of a short and long retention interval in cheater-memory studies [21], [29], no one has done so for the rarity strategy.

In sum, to test the cheater-memory versus rarity hypothesis, we had participants experience their computer partners' types in repeated Prisoner's Dilemma games. We varied the proportion of defectors and (conditional) cooperators among partners in a between-subjects design. Then, participants answered recognition and categorization questions in a memory test after 10 minutes and again after 1 week.

## Methods

### 2.1 Ethics Statement

The ethics committee of the Max Planck Institute for Human Development approved the study. Participants signed an informed consent before proceeding with the experiment.

### 2.2 Participants

Our lab recruited 126 participants (63 males, 63 females; mean [*M*] age = 26, range = 18–37, median [*Mdn*] = 26, mode [*Mo*] = 25) from the Berlin universities, 97 of which were students or in training. We excluded one participant for the analysis of the second session due to technical problems.

### 2.3 Stimuli and materials

For interaction partners, our design required 68 images of males and females with neutral facial expressions (participants and depicted volunteers were roughly of the same age). We used 58 color portraits from the database FACES ([30]; http://faces.mpib-berlin.mpg.de/album/escidoc:57488), with the volunteers wearing gray shirts, no make up or jewelery, sitting in front of a dark-gray background. For the remaining 10 images, we photographed students at the Technical University of Chemnitz in the same way as the FACES portraits. We randomly assigned popular German names (from the website http://www.beliebte-vornamen.de/) to the images for each participant. To avoid confusion about and interpretation of the faces, we informed the participants about the neutral character of the images.

We programmed and presented the experiment with E-Prime experimental software [31]–[32] (program available upon request). Participants received a written copy of the instructions during the experiment. The instructions contained the procedure of the interactions, illustrated with screenshots from the program (Document S1; original German instructions available upon request). In explaining the interactions in the instructions, we neither mentioned the Prisoner's Dilemma nor the words *game*, *payoff*, or *player*. Instead, we instructed participants that the aim of the experiment was to engage in a social interaction with a partner with whom they cannot correspond. An example illustrated this. The participants did not know about the memory task beforehand. As the final task, participants completed a questionnaire (Document S2) concerning their possible strategies and other remarks.

### 2.4 Procedure

The experiment involved two sessions separated by a mean of 7 days (range = 5–10 days, *Mdn* = 7, *Mo* = 7). The first session consisted of five phases and took approximately 80 min; the second session comprised four phases and lasted about 40 min.

#### 2.4.1 Session 1

After the participants had read the written instructions, we tested their understanding of the payoff matrix (Table 1) in the first phase of the session. We presented them with the four possible game outcomes (for example: “I cooperate, the partner refuses to cooperate.”) and asked them to indicate each time how many points they and their partner would receive according to the payoff matrix. They had to answer all situations correctly to continue to the next phase; otherwise, they repeated the phase (90 participants answered all four questions correctly after one round, 34 after two rounds, one after three rounds, one after six rounds).

**Table 1:** The payoff for the Prisoner's Dilemma game.

Player's Choice	Partner's Choice	
	Cooperate	Refuse
Cooperate	3 ; 3	0 ; 5
Refuse	5 ; 0	1 ; 1

*Note.* Payoff on the left in each cell is paid to the player, on the right to the partner.

In the second phase, participants practiced the interaction task by experiencing a series of Prisoner's Dilemma games in which they chose to cooperate or defect with each partner. The accumulated points, however, did not contribute to their final payment. Participants experienced four interaction partners whom they met for three interactions each (i.e., 12 encounters). Of the interaction partners, two were defectors (one male, one female) and two were cooperators (one male, one female). Afterwards, participants received feedback about their success (“You accomplished the practice session with [number] points profit. It would have been possible to achieve 14 to 24 points.”) and had the possibility for a short break.

The third phase was the actual interaction task in which we converted the payoff participants received into money. We randomly assigned participants to three conditions (42 participants in each condition) that differed in the proportion of partner types among the 20 computer partners. In the “defectors-rare” condition, 20% of interaction partners defected, 80% cooperated. In the “equal-proportion” condition, 50% defected, 50% cooperated, and in the “cooperators-rare” condition, 80% of partners defected and 20% cooperated. Each type comprised half male and half female partners. Whereas defector partners always defected, cooperator partners played Tit-For-Tat, which starts by cooperating and then copies the participant's previous action. Implementing a strategy that reacts to the participants' behavior maintains attention to the partner type. If participants faced a purely cooperative strategy, they might defect throughout to receive the highest payoff, losing the motivation to distinguish between the partner types. We informed participants that the partners were not human players but pursued strategies that had been identified in humans in experimental contexts before. They knew about neither the number nor nature of the partners' strategies.

**Figure 1:**
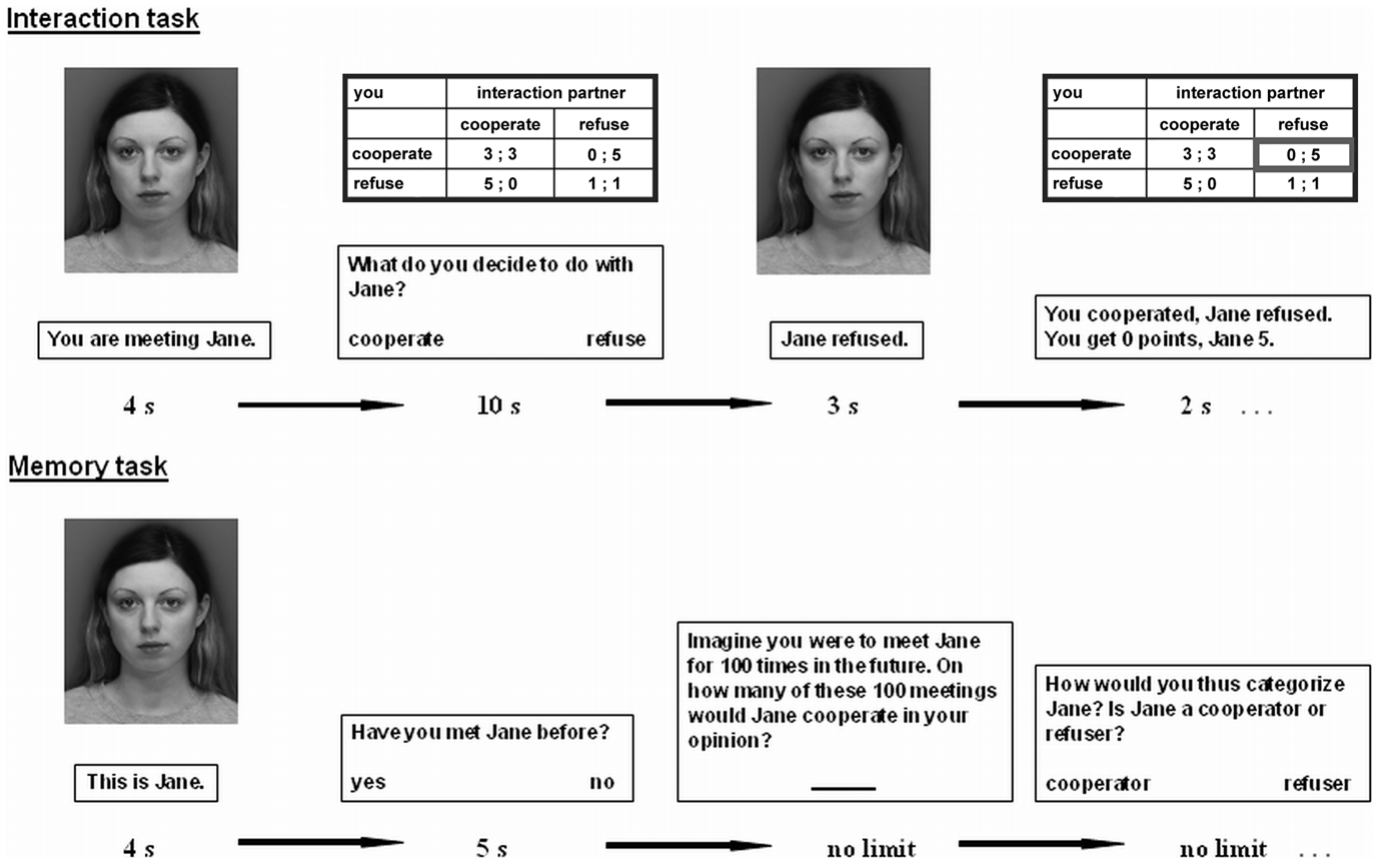
Example procedure of the interaction and the memory task. Screen presentation times are noted below. The original pictures were in color.

In the first block of interactions, participants met each of their 20 partners once. This procedure was repeated for 10 blocks, with a random order each time. Each encounter began with the presentation of the partner (Figure 1). After 4 s, the next screen asked participants to either cooperate or refuse to cooperate with the partner, and showed a picture of the payoff matrix. Participants had 10 s to respond; otherwise, this interaction was skipped and they were asked to answer more quickly next time. The subsequent screens displayed the decision of the partner for 3 s and finally gave a summary of the interaction for 2 s.

After a distraction task in which participants completed a shortened version of an episodic memory task [33]–[34] in 10–15 min, the final phase of the first session was the memory task (Figure 1). Here, participants saw images of the 20 old partners mixed with 20 new ones (half males, half females) and, for each partner, had to answer three questions. The first screen presented the partner for 4 s. Then, participants had 5 s to decide whether they had seen the partner before (recognition). They did not receive feedback on their success. Second, they rated the cooperativeness of the current partner on a scale from 0 (*no cooperative actions*) to 100 (*always cooperative*) and, third, categorized him or her as a defector or cooperator. On the latter two questions there was no time limit. Participants repeated this memory task for each partner.

#### 2.4.2 Session 2

After a mean of 7 days, participants returned for the second session. They began by reading the written instructions and then proceeded with the memory task. We presented participants with the 20 old partners from the first experimental session and 20 new partners (half males, half females) they had not seen in any phase before. The procedure of the memory task was the same as in the first session.

Afterwards, participants had the opportunity for a short break and then experienced three Prisoner's Dilemma games with 20 partners from the memory task. Half of these partners were old, the other half participants had not seen before. The proportion of types among the partners conformed to the conditions like in the first session, and, again, each type comprised half male and half female partners. The procedure of the interactions was the same as in Session 1. Then, participants completed the distraction task, and, in the final phase, they answered questions concerning their strategies in the two sessions. Finally, participants received 5 euro show-up fee per session. Additionally, we paid participants according to the overall points received in the interaction task in both sessions by multiplying their gains with 0.02, 0.03, and 0.06 euro in the defectors-rare, equal-proportion, and cooperators-rare condition to equate the total payment across conditions (range_Defectors Rare_ = 7.98–13.40 euro; range_Equal Proportion_ = 10.11–13.86 euro; range_Cooperators Rare_ = 9.36–19.56 euro). Although participants received different numbers of points due to the proportion of partner types in the conditions, this did not influence the absolute number of partners correctly recognized and categorized. Participants did not know about the different exchange rates when making their choices and categorizations, so this could not influence the results.

### 2.5 Design and data analysis

As the independent variable, we varied the proportion of defectors and cooperators in the interaction group in a between-subjects design (defectors rare, equal proportion, cooperators rare). As the main dependent variables, we assessed recognition and categorization judgments, as well as a quantitative cooperativeness evaluation for each partner. Moreover, we collected choice data – the participants' proportions of defection against and cooperation with partners – to check whether participants were able to distinguish between the partner types. With descriptive statistics, we present mean, standard deviation, median, and mode; for comparisons between proportions, we give the mean with 95% confidence interval (e.g., [35]) and Cohen's [36] *h* effect size (Cohen's conventions: small effect size: *h* = 0.20, medium effect size: *h* = 0.50, large effect size: *h* = 0.80). If the proportions are compared to chance performance at 50% (i.e., 0.5), we report Cohen's *g* (Cohen's conventions: small effect size: *g* = 0.05, medium effect size: *g* = 0.15, large effect size: *g* = 0.25). When comparing results between sessions or repetitions, we accounted for within-subject variation by applying Morey's [37] correction of Cousineau's [38] transformation for confidence intervals. To evaluate the recognition performance, we provide Snodgrass-Corwin-corrected *d*' measurements (e.g., [39]).

We looked for the cheater-memory and rarity strategy in the categorization accuracy in conjunction with correct recognition of old partners, because also in everyday life one must do both—correctly recognize and correctly categorize to sufficiently identify a partner. In the memory research literature, this measure is called SIM, source identification measure [40], and is calculated as the number of correct categorizations given correct recognition over the number of all answers (correct recognition and correct categorization, correct recognition and incorrect categorization, incorrect recognition) for each partner type. Because participants make errors and guess when categorizing partners, the data analysis should account for guessing biases [9]–[10], [28]–[29]. We calculated chance levels, that is, the accuracy achieved by guessing, as the proportion of categorized defectors and cooperators any time participants recognized a partner, whether old or new, as old and corrected the raw accuracy rates for these chance levels. Data are available in Table S2.

## Results

### 3.1 Exclusion of participants

Participants who almost never cooperated experienced only minimal cooperation by Tit-For-Tat partners and, thus, could hardly distinguish between the partner types. From these participants, we did not expect proper partner identification in the memory task. We excluded 27 participants (four, 11, and 12 participants from the three conditions) who cooperated with Tit-For-Tat partners in at most 13% of the cases. At this percentage, there seemed to be a natural gap in the data. The next nearest value of “percentage of cooperation with cooperator partners” in the defectors-rare, equal-proportion, and cooperators-rare condition was at 18%, 27%, and 20%. All further analyses, therefore, used only the data from the remaining 99 participants. By excluding 27 participants, the mean proportion of cooperation with defector and cooperator partners increased, specifically for the equal-proportion and cooperators-rare condition. Moreover, the mean cooperativeness evaluation of cooperator partners increased. The categorization accuracy increased, specifically for the equal-proportion and cooperators-rare condition. All in all, however, by excluding the 27 participants, the results did not change dramatically. Additionally, we excluded cooperativeness evaluations from one participant in the defectors-rare condition in both sessions who seemed to have misunderstood the task and evaluated partners categorized as defectors with values around 96.2 (*SD* = 7.4) and partners categorized as cooperators with values of 1 (*SD* = 0).

### 3.2 Cooperative behavior

#### 3.2.1 Session 1

**Figure 2:**
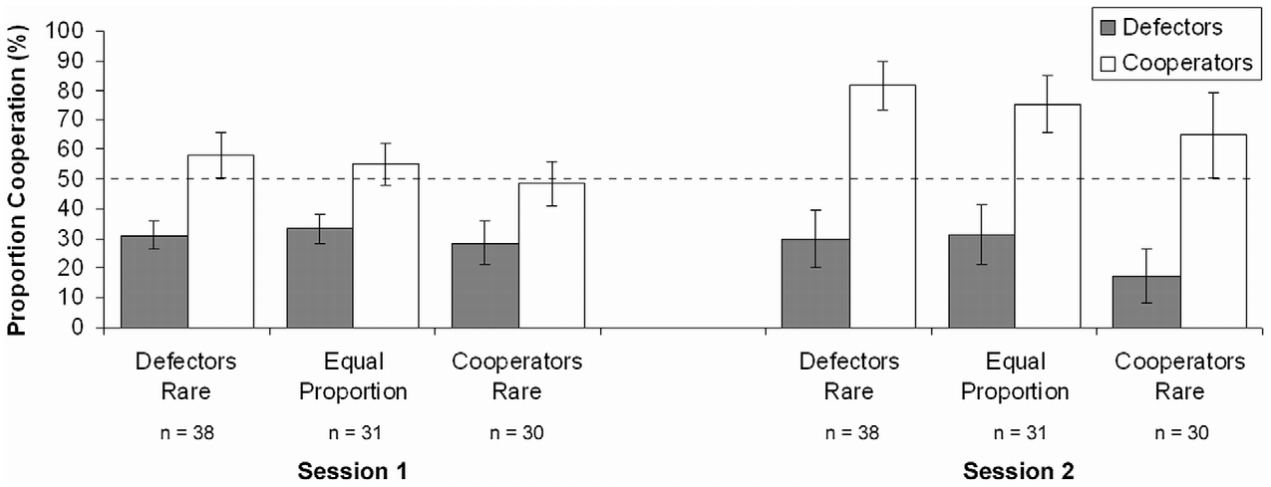
Proportion of cooperation with defector and cooperator partners. The dashed line represents chance performance. In the defectors-rare condition, of the 20 interaction partners 20% were defectors and 80% cooperators (Tit-For-Tat). The equal-proportion condition included 50% defectors and 50% cooperators, and the cooperators-rare condition included 80% defectors and 20% cooperators. The *n*s give the number of participants per partner type. Error bars represent 95% confidence intervals.

Participants experienced 10 Prisoner's Dilemma interactions with each partner. To maximize their payoff (Table 1), participants should defect against a defector partner and cooperate with a cooperator partner. Whereas participants cooperated with defectors less than expected by chance in all conditions, they cooperated with cooperators more than expected by chance only when cooperators were common (in the defectors-rare condition; *g* = 0.08; Figure 2) and when both partner types had an equal proportion (*g* = 0.05). Yet, we found more cooperation with cooperators than defectors in all conditions, and participants cooperated more with cooperators when they were common (in the defectors-rare condition; *M* = 58.0%±7.7 CI) than when they were rare (*M* = 48.5%±7.4 CI; *h* = 0.20; no difference between the other conditions).

**Figure 3:**
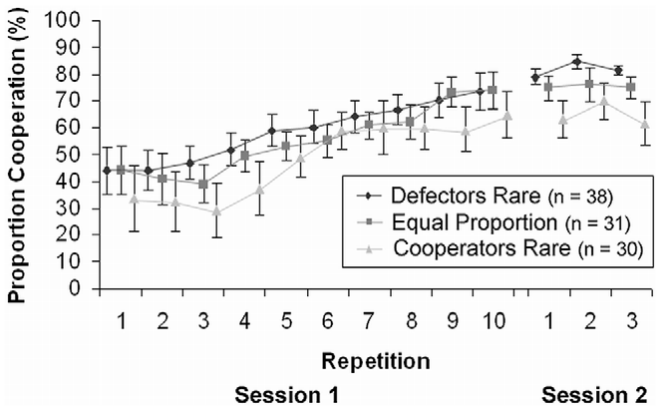
Proportion of cooperation across repetitions. The figure shows the mean proportion of cooperation (±95% confidence intervals) with cooperator partners across the repeated interactions in the first and second session. Confidence intervals are corrected to account for within-subject variation (Morey, 2008).

We did not expect these low rates of cooperation with cooperator partners, but the analysis across the 10 repetitions revealed that the mean cooperative behavior increased from 40.8%±5.8 CI in the first round to 71.0%±4.3 CI in the tenth round (Figure 3).

#### 3.2.2 Session 2

Figure 2 shows participants' cooperative behavior, averaged across the three interactions, in the second session after about 1 week. This time, participants cooperated both with defectors less than expected by chance and with cooperators more than expected by chance in all conditions (g_Defectors Rare_ = 0.32, g_Equal Proportion_ = 0.25, g_Cooperators Rare_ = 0.15). They seemed to distinguish between the partner types and acted accordingly. Consequently, the proportion of cooperation with cooperators in the beginning of the second session (*M* = 72.9%±3.2 CI) was at the level of the tenth repetition in the first session (*M* = 71.0%±4.3 CI; Figure 3).

### 3.3 Recognition

#### 3.3.1 Session 1

In the memory task, we first asked participants whether they had already interacted with each of 40 partners (20 old, 20 new ones). Participants recognized the 20 old partners accurately (*M_hit rate_* = 99.1%, *SD* = 2.5, *Mdn* = 100, *Mo* = 100) and showed low false alarm rates (false alarms/[false alarms+correct rejections]; *M_false alarm rate_* = 0.6%, *SD* = 1.7, *Mdn* = 0, *Mo* = 0; *d'* = 3.8)—they distinguished between old and new partners quite well.

#### 3.3.2 Session 2

In the second session, participants showed high accuracy (*M_hit rate_* = 98.3%, *SD* = 5.1, *Mdn* = 100, *Mo* = 100) and low false alarm rates (*M_false alarm rate_* = 0.8%, *SD* = 3.7, *Mdn* = 0, *Mo* = 0; *d'* = 3.6), suggesting excellent recognition even after a one-week retention interval.

### 3.4 Cooperativeness evaluation

**Figure 4:**
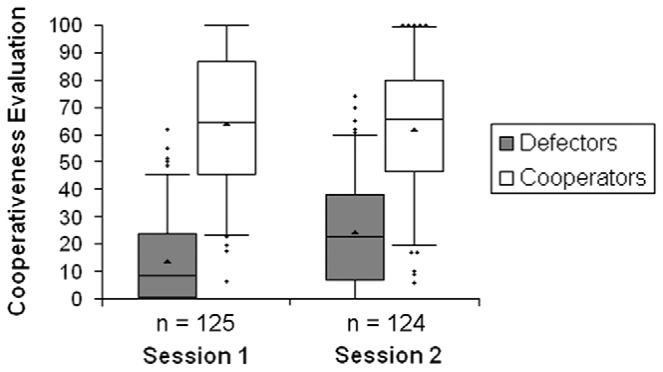
Cooperativeness evaluations of defector and cooperator partners. Boxplots represent cooperativeness evaluations of the partner types among old partners for both sessions. Boxplots show the median as a line inside the box, which contains 50% of the data (upper border = 75^th^ percentile, lower border = 25^th^ percentile). The triangle represents the mean. The whiskers range from 5 to 95% of the data, outliers are represented as diamonds. We additionally excluded the data from one participant in the defectors-rare condition in both sessions who seemed to have misunderstood the task.

The second question of the memory task evaluated the cooperativeness of partners on a scale from 0 (*no cooperative actions*) to 100 (*always cooperative*). Overall, defector partners among the old partners received low cooperativeness values and cooperator partners received high cooperativeness values in both sessions, matching the strategies of the partner types (Figure 4). The larger variability for cooperator partners in each session reflects the reactivity of the Tit-For-Tat strategy to the participants' behavior (cooperator partners' *M_cooperation rate_* = 57.2%, *SD* = 19.9, *Mdn* = 55, *Mo* = 50).

### 3.5 Categorization

#### 3.5.1 Session 1

We analyzed the results of the memory task as the accuracy of categorization in conjunction with correct recognition of old partners. This accuracy rate, however, must be corrected for the chance level of accuracy reached by participants' guessing the answers. To represent the chance level, we considered the perceived proportion of defectors and cooperators among all partners, whether old and new, recognized as old. To correct for chance performance, we present the simple difference between accuracy rate and chance level, averaged across participants.

**Figure 5:**
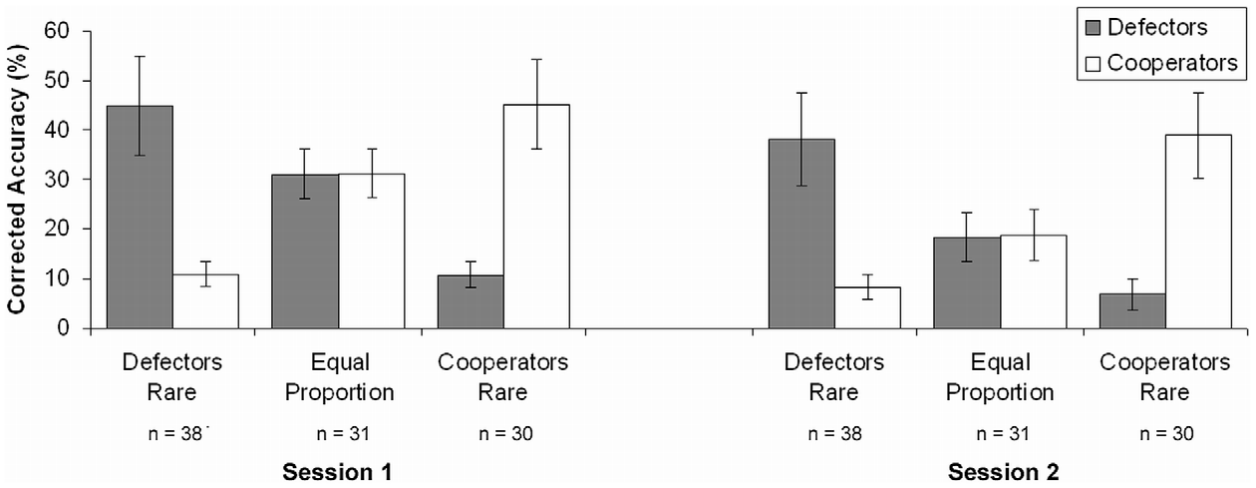
Accuracy rates for defector and cooperator partners. For the depicted accuracy rates, we calculated categorization accuracy in conjunction with correct recognition of old partners per participant, subtracted individual chance levels (i.e., the perceived proportion of partner types among old and new partners recognized as old), and averaged across participants. Error bars represent 95% confidence intervals.

We found that, on average, defectors were remembered better than cooperators in the defectors-rare condition (*h* = 0.80), cooperators remembered better than defectors in the cooperators-rare condition (*h* = 0.80), and both partner types were remembered equally well in the equal-proportion condition (Figure 5). This matches the predictions of the rarity hypothesis. Analyzing the data at the individual's level showed that this pattern held for most of the participants: 89% of participants in the defectors-rare condition remembered defectors better than they remembered cooperators, 93% in the cooperators-rare condition remembered cooperators better than they remembered defectors, and 84% in the equal-proportion condition remembered both partner types equally well.

#### 3.5.2 Session 2

Correcting the accuracy rate for the chance level per person in Session 2 resulted, on average, in defectors being remembered better than cooperators when defectors were rare (*h* = 0.75), cooperators remembered better than defectors when cooperators were rare (*h* = 0.80), and both partner types remembered equally well when they were equally common (Figure 5). This was true for most participants: 92% in the defectors-rare condition remembered defectors better than they remembered cooperators, 93% in the cooperators-rare condition remembered cooperators better than they remembered defectors, and 77% in the equal-proportion condition remembered both partner types equally well. Again in Session 2, the accuracy rate corrected for the chance level supported the predictions of the rarity hypothesis. Across sessions, the accuracy corrected for chance slightly decreased for cooperator and defector partners in all conditions.

## Discussion

To explore whether people better remember cheaters or the rare partner type, we varied the proportion of defectors and cooperators (represented by Tit-For-Tat partners) in the interaction group in a between-subjects design and tested whether a cheater-memory or a rarity strategy matched the accuracy rates of partner categorization in conjunction with correct recognition better. Accounting for the perceived proportion of partner types in each condition, in the short (after 10 min) and long run (after 1 week), participants remembered the rare partner type in the interaction group better than they remembered the common one. This pattern of results matches the predictions of the rarity hypothesis.

Our study extends the work on the rarity strategy in cooperation [9]–[10] by addressing two issues: the role of experience and long-term memory retention.

### 4.1 Does experience influence categorization?

Rather than reading about their partners' behavior, our participants experienced the partner types in repeated interactions. This way, they could form their own impressions, were personally involved by receiving the payoffs from these interactions, and had the opportunity to establish reciprocal relationships in a more ecologically valid situation. Cooperation with the cooperators increased over the course of 10 repetitions (Figure 3), confirming that participants require the repeated interactions to become acquainted with the partner types. This resulted in high recognition and categorization rates (even after a week). The recognition rates we observed are higher than those in previous studies on partner-type memory by 13–65% (Table S1). Compared to other studies collecting categorization accuracy in conjunction with correct recognition of old partners (Barclay, personal communication; [20], [22]), our conjunction-categorization rates exceed the rates of these studies by 8–68% (Table S1). Some studies reported categorization accuracy of old partners independent of correct recognition (e.g., [9]). If we calculate this independent categorization accuracy (Figure S1), our rates differ by −11–50% (Table S1). Remembering the moves from repeated interactions with partners whose strategies react to one's own behavior seems to be a difficult task—more difficult than remembering one move per partner as in the previous studies. Nevertheless, repetition pays off by allowing a stronger encoding of partners and an accurate summary of their behavior. The assignment of types allows individuals to ignore occasional defections of cooperator partners, for example, as long as the cooperator partners, in general, cooperate. This robustness towards variance in the partner's behavior also applies to memory errors, which makes the assignment of types a good strategy with high memory load. To accurately categorize partners, it seems, repeated interactions are an important component.

### 4.2 How robust are the memory strategies to longer retention intervals?

Barclay [9] and Bell et al. [10] found support for the rarity strategy in a memory test minutes after the presentation of the partners. We confirmed this finding and replicated the result in a memory test 1 week after the initial presentation. So, despite this long retention interval, participants performed similarly in the first and second session in correctly recognizing previously seen partners and categorizing defectors and cooperators. This is consistent with the idea that categorizing partners into types is a stable criterion that lasts longer than an immediate repeated encounter. Though accuracy levels decreased slightly across the sessions, this did not seem to interfere with the rarity strategy—approximately the same number of participants showed a preferential memory for the rare partner type in the first and second session. This means that the relation between categorization rates (in conjunction with correct recognition) and chance levels used to correct these rates must be similar. As the chance levels represent participants' perception of the proportion of partner types, one can conclude that not only is participants' memory for partner types robust to longer retention intervals but also their memory for the environment. Compared to other studies collecting categorization accuracy in conjunction with correct recognition of old partners, our conjunction-categorization rates from the second session after 1 week exceed the rates from studies with retention interval of several minutes by 10–47% (Table S1). Some studies reported categorization accuracy of old partners independent of correct recognition (e.g., [9]). If we calculate this independent categorization accuracy (Figure S1), our rates from 1 week retention differ from the study with several minutes retention by −8–37% (Table S1). So, our results from the long retention interval of 1 week even mostly exceed those results from studies with several minutes retention, emphasizing the unusual robustness of our categorization results. Moreover, participants benefited from their experience with and improved knowledge of the partner types, indicated by a comparable proportion of cooperation with cooperators in the beginning of the second session as in the end of the first session (Figure 3). These findings speak to the importance of memory as one of the prerequisites for establishing long-lasting social interactions via reciprocity and promoting the emergence of cooperation.

### 4.3 Alternative analytical methods

Additionally to addressing the two questions mentioned above, our study, compared to previous ones, employed a different analytical method. Would we still find a rarity effect if we analyzed the data with previously used methods (Document S3)?

Compared to Barclay [9], our method differs in three aspects—the accuracy rates to test the hypotheses on, the chance level for which to account the accuracy rates, and the way how to account for the chance level. First, whereas Barclay investigated the cheater-memory and rarity strategy in the categorization accuracy independent of correct recognition, we calculated the categorization accuracy in conjunction with correct recognition of old partners, because we consider this a necessary requirement for partner categorization in everyday life. So, as opposed to Barclay, our accuracy rates do not contain categorizations of old partners falsely recognized to be new. Second, that is why, for chance level, we did not take into account the perceived proportion of partner types among old and new partners, like Barclay did, but the perceived proportion among old and new partners recognized as old. Third, Barclay calculated the difference between accuracy rate and chance level relative to the individual chance levels using [(accuracy – chance level)/chance level], but this relative correction biases the difference score in favor of the rare type, increasing the probability of finding a rarity effect. We subtracted the individual chance levels from the accuracy rates to yield a less biased measure. So, our method constitutes categorization accuracy in conjunction with correct recognition of old partners, a chance level of the perceived proportion of partner types among old and new partners recognized as old, and the correction for chance performance by taking the simple difference between accuracy and chance level. Barclay's method constitutes categorization accuracy for old partners independent of correct recognition, the perceived proportion of partner types among old and new partners, and the correction for chance by taking a relative difference. Regardless of the method applied, though, our data always produce a rarity effect (Figure S1).

Bell et al. [10] analyzed their data with the aid of multinomial processing tree (MPT) models. This method distinguishes recognition, categorization, and various guessing biases [41]. Employing a model by Bayen, Murnane, and Erdfelder [42], Bell et al. [10] found a rarity effect also in a reanalysis of Barclay's [9] data. Though we attempted an analysis with our data, we could not apply the MPT method. One guessing assumption MPT models incorporate is the categorization of new partners falsely recognized as old, but participants in our study discriminated old from new partners so accurately that we only had few data points for this category in all conditions and sessions. With scarce data for this guessing assumption, the MPT model could not produce precise estimates for our categorization parameters. Although scarce data distort the analysis with MPT models, this is not a disadvantage of the study. We believe our participants discriminated old from new partners so well, because they were acquainted with them through the repeated interactions. This large amount of experience offers a more realistic situation compared to meeting the partner once, like in a one-shot game. Therefore, the proportion of new partners falsely recognized as old, alone, may not be an appropriate guessing assumption for data with high recognition.

### 4.4 Limitations

The design of our study is limited in some ways that could potentially influence the results. First, rather than cooperating unconditionally, our cooperator partners played Tit-For-Tat and, therefore, were not as easily identifiable as cooperators. Frequently defecting participants did not experience much cooperation by cooperator partners and might not realize that these partners are cooperators, reflected by the large variability of cooperativeness evaluations of cooperator partners (Figure 4). Experiencing cooperator partners as cooperative, however, is crucial for categorizing them correctly and can otherwise decrease categorization accuracy. The alternative, implementing unconditionally cooperating partners, could have resulted in greater disadvantages. Participants might defect with these pure cooperators, because there are no costs of exploiting them. In effect, participants would lose the motivation to track partner types which potentially would have decreased categorization accuracy.

Second, whereas letting participants take part in a game increases commitment, a Prisoner's Dilemma game offers only a limited model of cooperative interactions outside the lab. This limits the generalizability of our results. First, according to the definition by Cartwright ([43], p. 86), reciprocal altruism is time-delayed mutualism—donor and recipient of cooperative acts alternate in their roles so that there passes a certain amount of time between the tit and the tat. In the Prisoner's Dilemma we used, however, the exchange of actions happened simultaneously so that a participant was donor and recipient at the same time. Thus, using a sequential Prisoner's Dilemma that enables the alternation of the roles might have modeled the situation we actually wanted to investigate more closely [44]–[45]. Second, the setup of the game has to be chosen with care: The payoff matrix can influence the behavior of participants [46], and using computer instead of human partners affects participants as well [47].

### 4.5 Conclusion

Our study confirms evidence of a general strategy to remember the rare interaction partners—also in repeated encounters, over a long retention interval, and regardless of the analytical method applied. We reject the cheater-memory hypothesis: In this study, cheaters are not remembered preferentially regardless of the environment. Given that the rarity and cheater-memory hypotheses make the same predictions when defectors in the interaction group are rare, the cheater-memory strategy could be the implementation of the rarity strategy in this kind of environment, though. Contrary to always remembering the same partner type (i.e., cheaters) regardless of the environment, however, the strategy seems to be to remember the type that is rare in the respective environment. Our findings, thereby, support the idea of a cognitive architecture that flexibly responds to the environment instead of specializing in certain interaction groups. By applying the toolbox metaphor of the “fast and frugal heuristics” program [48], our findings suggest that rather than using the same tool (i.e., remember the same partner type) in all possible environments, participants responded in an ecologically rational way by remembering partner types differentially depending on the environment [15].

Moreover, our results have implications for the design of new strategies to explain the emergence of cooperation. The traditional reciprocal strategies such as Tit-For-Tat require remembering the partners' single last action and do not distinguish in memory accuracy between defection or cooperation behavior. Stevens et al. [7] showed that, when asked to remember the single last action, individuals do not preferentially remember cooperation or defection. Our results, on the other hand, indicate that memory can differentiate between the behavior in partner types. The combination of these findings leads the way to more realistic strategies that store partner types instead of single actions and distinguish in memory accuracy between defectors and cooperators depending on the environment. Research on indirect reciprocity has already produced strategies acting on the partner's reputation as acquired in the interactions with third parties [49]–[50]. In evolutionary simulations, these strategies outcompeted their opponents and promoted the evolution of cooperation.

In sum, repeatedly meeting interaction partners seems to improve partner-type memory, as we found higher recognition and categorization (dependent and independent of correct recognition) rates compared to previous studies. This strong encoding of partner types could explain the high accuracy rates and the robustness of the memory strategy even after a retention interval of 1 week. Our results suggest that the rarity of defectors and cooperators in the environment influence how well they are remembered. It looks as if people indeed try to minimize costs—not the costs associated with exploitation, as suggested by the cheater-memory hypothesis, but the costs associated with memory errors. Of two people with whom you interacted, the cheater might be the more important partner type to remember, but in an environment where cheaters represent the majority, the costs for remembering all of them overrule the costs of exploitation.

## Supporting Information

Document S1 **Instructions for the first session.**
(DOC)

Document S2 **Questionnaire.**
(DOC)

Document S3 **Categorization using Barclay's method.**
(DOC)

Figure S1 **Accuracy rates using Barclay's method.** Part (a) depicts the categorization accuracy for old partners independent of correct recognition (±95% confidence interval) in the three conditions in the first session. The solid line represents the chance levels based on the perceived proportion of partner types among old and new partners. In part (b), we present the relative differences between accuracy rates and chance levels using [(accuracy rate – chance level)/chance level] averaged across participants. The lower parts (c and d) show the respective results from the second session after 1 week. In the cooperators-rare condition, we averaged across *n* = 30 for defectors and *n* = 29 for cooperators in each session.
(TIF)

Table S1 **Accuracy rates for recognition, categorization independent of correct recognition, and categorization in conjunction with correct recognition (with 95% confidence intervals) from different studies investigating partner-type memory.** Note. We only report partner-memory studies that provided raw values in the paper. * We calculated the 95% confidence interval from the standard deviations given. † SIM = Source Identification Measure. ‡ The values give the range of results for studies distinguishing several conditions or separating participants by gender.
(DOC)

Table S2 **Raw data.**
(XLS)
