# Supplementary material for: The Good, the Bad, and the Rare: Memory for Partners in Social Interactions
Source: PLoS One. 2011 Apr 29;6(4):e18945. doi: 10.1371/journal.pone.0018945 (PMC3084729; doi:10.1371/journal.pone.0018945)
Supplement: Document S1 — Instructions for the first session. (DOC) [file pone.0018945.s001.doc]

Document S1. **Instructions for the first session**

This experiment is about social interactions. You will repeatedly interact with other people. Depending on what your interaction partner and you decide to do, you will receive points. These points will be converted into money which you will be paid in the end. Your interaction partners are not actually people but they pursue strategies that have been identified in humans in experimental contexts before.

The interaction

The interaction is about agreeing with the opponent without being able to talk to one another. Imagine, for example, you produced some work in collaboration with a colleague. Your boss is not satisfied with the quality and calls the two of you individually into his office to search for reasons and maybe find the one to blame. Further, imagine your colleague and you just have the choice between “cooperating” or “refusing to cooperate” with the other. “Cooperating” in this case means to remain silent; “refusing” is to blame the other one. Even if your colleague was at your boss’ office first, you do not have a chance getting to know what he decided to do before you go in there yourself—you do not have the opportunity to talk to one another. Depending on what your colleague and you decide to do, there arise 4 possibilities:

1. You refuse to cooperate with your colleague and blame him, whereas he aims to cooperate and remains silent. Thereby, you are looking pretty good in the eyes of your boss; your colleague attracts the whole resentment.

2. Your colleague and you refuse to cooperate with each other and blame one another. The boss will think that none of you is completely innocent when it comes to the quality of the work and call both of you to account.

3. Your colleague and you cooperate and remain silent what concerns the one to blame. Your boss will be insecure and teach both of you at least a little lesson.

4. You protect your colleague and remain silent, whereas he blames you. You will have to carry the whole damage yourself, whereas your colleague gets away without a penalty.

In the experiment, these different results are translated into points you earn, depending on what your partner and you decide to do. You see the distribution of points in Table 1.

Table 1

Payoff in points for all interaction situations.

| You | Interaction Partner | |
| --- | --- | --- |
|  | Cooperate | Refuse |
| Cooperate | 3 ; 3 | 0 ; 5 |
| Refuse | 5 ; 0 | 1 ; 1 |

Please have a close look at the payoff matrix: You cannot just see the points you will receive for all of the four situations (left value in each cell) but also those that your partner will get (right value in each cell). If you decide, for example, to “refuse”, the other person to “cooperate”, the lower left cell comes into effect: You receive 5 points, your interaction partner 0. If both of you “refuse”, each one of you earns 1 point (lower right cell). If the two of you opt for “cooperate”, each of you will get 3 points (upper left). If you choose “cooperate”, your partner “refuse”, you will get 0 points, your partner 5 (upper right).

The procedure

First, the partner will be introduced to you with image and name (Please note that the people on the images were asked to look neutrally. Jewelry and possible make-up were removed. They all wear the same t-shirt.). In the example below, your partner is Bernd.


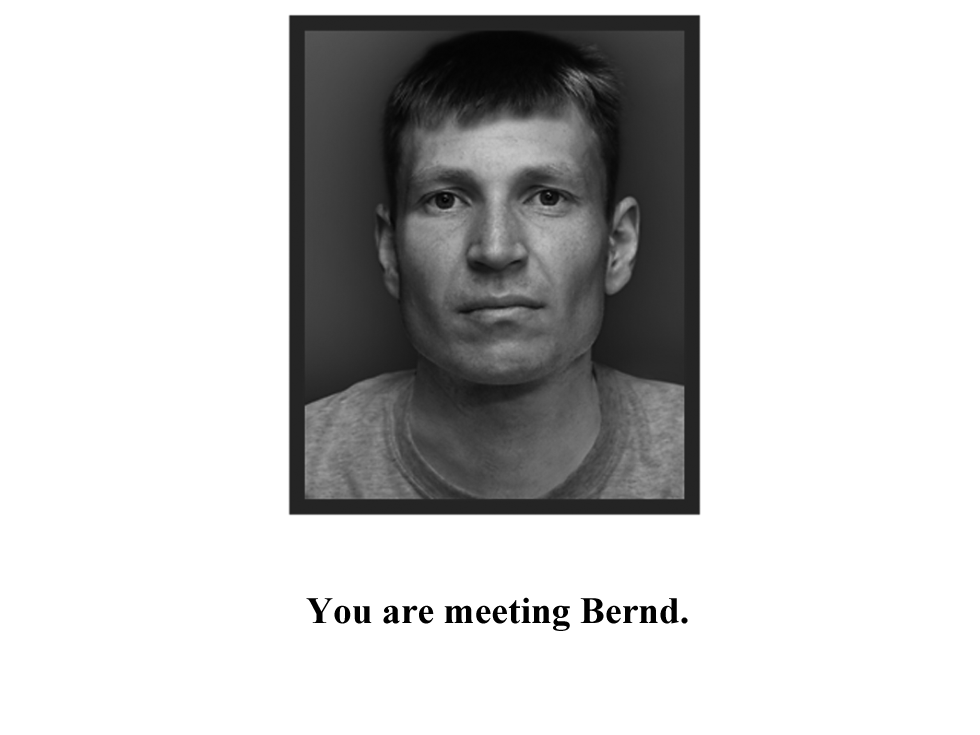


After that, you will be asked to choose one of two alternatives. To do so, please press the key “q” for “cooperate” or “p” for “refuse”. You will have ten seconds to react. If you wait longer than ten seconds, the question will be skipped.


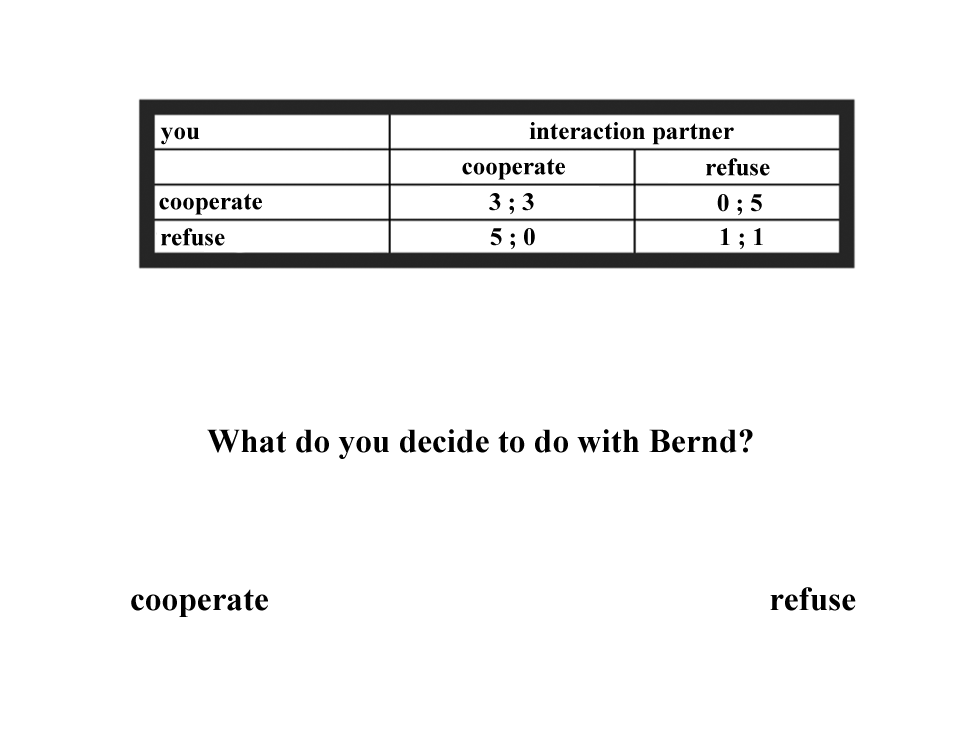


Assume you chose “cooperate”. Imagine your interaction partner (here: Bernd) got the question what to do with you at the same time. On the next screen (and in the example below) you will experience what the interaction partner decided to do.


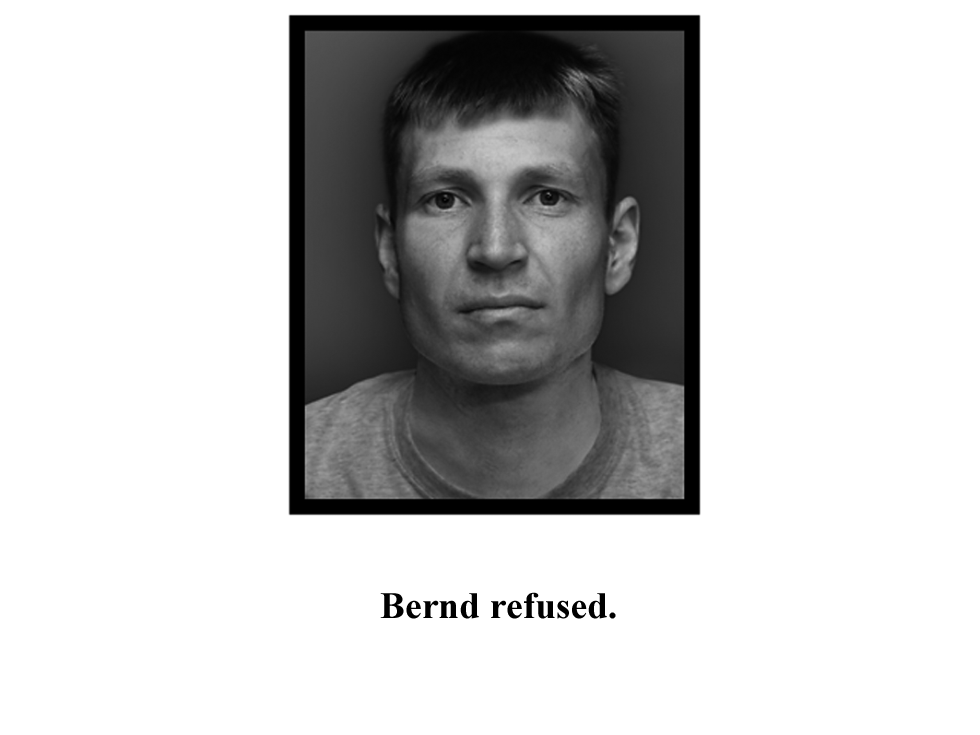


Next, you will be shown with the help of the payoff matrix how many points you and your partner receive. In this case, you will get 0 points, Bernd 5.


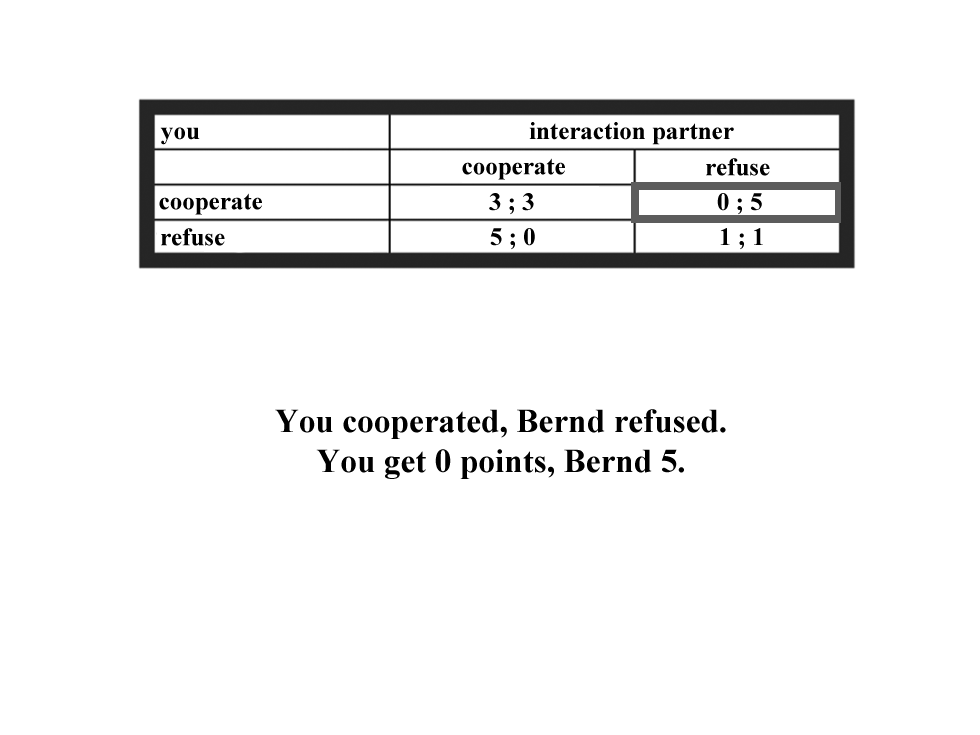


Therewith, the interaction with Bernd is over. What follows is introduction, decision question, partner decision, and payoff information for the next partner and so on. After you have interacted with each person, you will meet all of them again repeatedly and in random order.

A certain percent of the overall number of points you will be paid in the end (additionally to the 5 euro show-up fee). All in all, you may earn approximately 4-16 euro additionally. You will not earn money for skipped questions.

The overview

The experiment begins with a phase which will test whether you understood the payoff matrix. You will only continue when you answered more than 80% of the questions correctly. After that, there follows a practice phase in which you will get to know the interaction situation without earning money. Then, the actual interactions will take place. Thereupon, two tasks are attached for which instructions will be given on the screen. The whole session should take about 80 min.

As the experimenter already told you, this experiment requires an additional session (approximately 40 minutes) in a week. You absolutely have to participate in this; the date cannot be postponed. Details concerning the procedure and the tasks will be given to you then.

Should you have any questions, please ask the experimenter. If you are ready to begin with the first phase, please press the space bar on the keyboard.

Have fun with the experiment and thanks for your collaboration!
