# Supplementary material for: The Good, the Bad, and the Rare: Memory for Partners in Social Interactions
Source: PLoS One. 2011 Apr 29;6(4):e18945. doi: 10.1371/journal.pone.0018945 (PMC3084729; doi:10.1371/journal.pone.0018945)
Supplement: Document S2 — Questionnaire. (DOC) [file pone.0018945.s002.doc]

Document S2. **Questionnaire**

1. Did you pursue a certain strategy in interacting with the partners? If yes, please describe the strategy you used.

2. In case you memorized cooperators/refuser in particular: Why did you choose the one or the other group?

3. How did you come to a decision when you could not remember your partner’s action from the last interaction?

4. How much were you involved emotionally in the experiment?


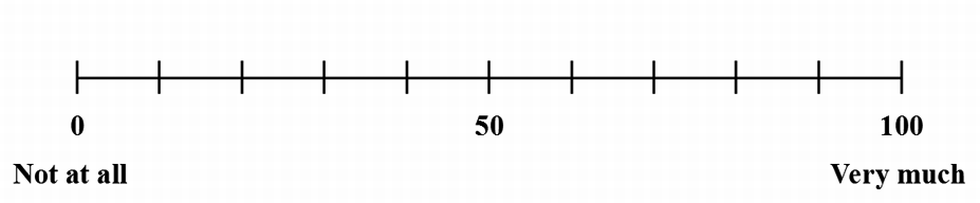


5. Were you angry about something? If yes, what was it?

6. How angry were you on a scale from 0 (*not at all*) until 100 (*very much*)?


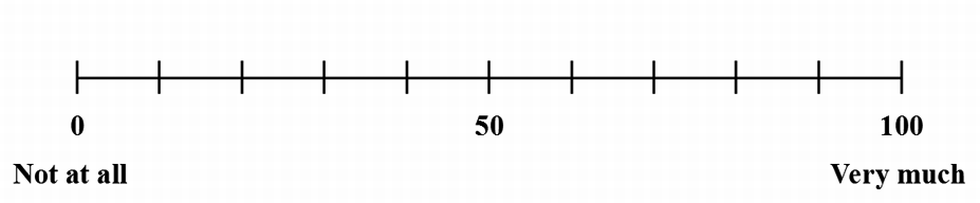


7. Were you happy about something? If yes, what was it?

8. How happy were you on a scale from 0 (*not at all*) until 100 (*very much*)?


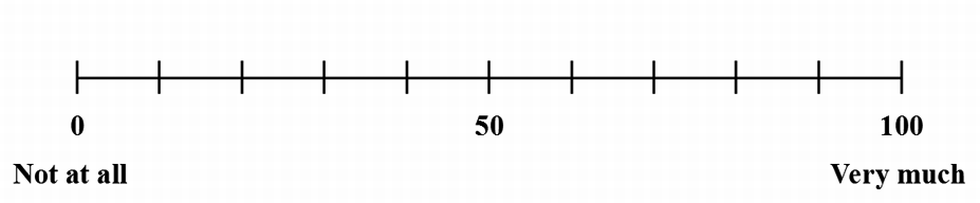


9. Do you know one/some of the depicted persons? If yes, which one/ones?

10. Did you associate the memory of a specific person/specific persons with one/some of the names? If yes, with which name/names?

11. Did you notice differences between the pictures (color of the background/shirt, light, quality)? If yes, which pictures differed?

12. How interesting did you find the whole study (Session 1 and 2) on a scale from 0 (*not at all*) until 100 (*very much*)?


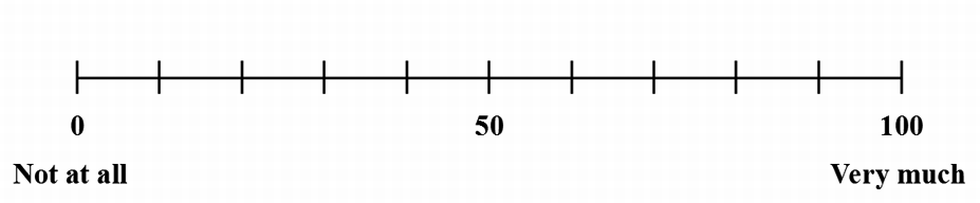


13. If you have comments or suggestions concerning the experiment, please note them.
