# Supplementary material for: The Good, the Bad, and the Rare: Memory for Partners in Social Interactions
Source: PLoS One. 2011 Apr 29;6(4):e18945. doi: 10.1371/journal.pone.0018945 (PMC3084729; doi:10.1371/journal.pone.0018945)
Supplement: Document S3 — Categorization using Barclay's method. (DOC) [file pone.0018945.s003.doc]

Document S3. **Categorization using Barclay’s method**

To compare our findings to Barclay’s [9], we applied his analytical method to our data. For accuracy rates, he considered the correct categorizations of the 20 old partners, independent of correct recognition (Figure S1a). For chance level, he proposed the perceived proportion of partner types among old and new partners. To correct the accuracy rates for the chance levels, Barclay used a relative difference [(accuracy rate – chance level) / chance level] and averaged across participants. We excluded data from 3 participants due to division by 0. Applying Barclay’s method to our data, we found that in the defectors-rare and equal-proportion condition defectors were categorized more accurately than cooperators; in the cooperators-rare condition, cooperators were categorized more accurately than defectors (Figure S1b). When looking at individual participants, the majority of them showed the pattern in the respective condition: 97% of participants in the defectors-rare and 77% in the equal-proportion condition categorized defectors better than they categorized cooperators, 66% in the cooperators-rare condition categorized cooperators better than they categorized defectors.

We observed a similar pattern of results in the second session (Figures S1c, d). The individual data analysis showed that this held for most of the participants: 83% in the defectors-rare condition and 61% in the equal condition categorized defectors better than they categorized cooperators, 66% in the cooperators-rare condition categorized cooperators better than they categorized defectors.

In both sessions, the results, given the chance levels of perceived proportion of partner types among old and new partners, support the predictions of the rarity hypothesis in that participants better remember the partner type that is rare in the interaction group.
