# Supplementary figures and images for: The Good, the Bad, and the Rare: Memory for Partners in Social Interactions
Source: PLoS One. 2011 Apr 29;6(4):e18945. doi: 10.1371/journal.pone.0018945 (PMC3084729; doi:10.1371/journal.pone.0018945)

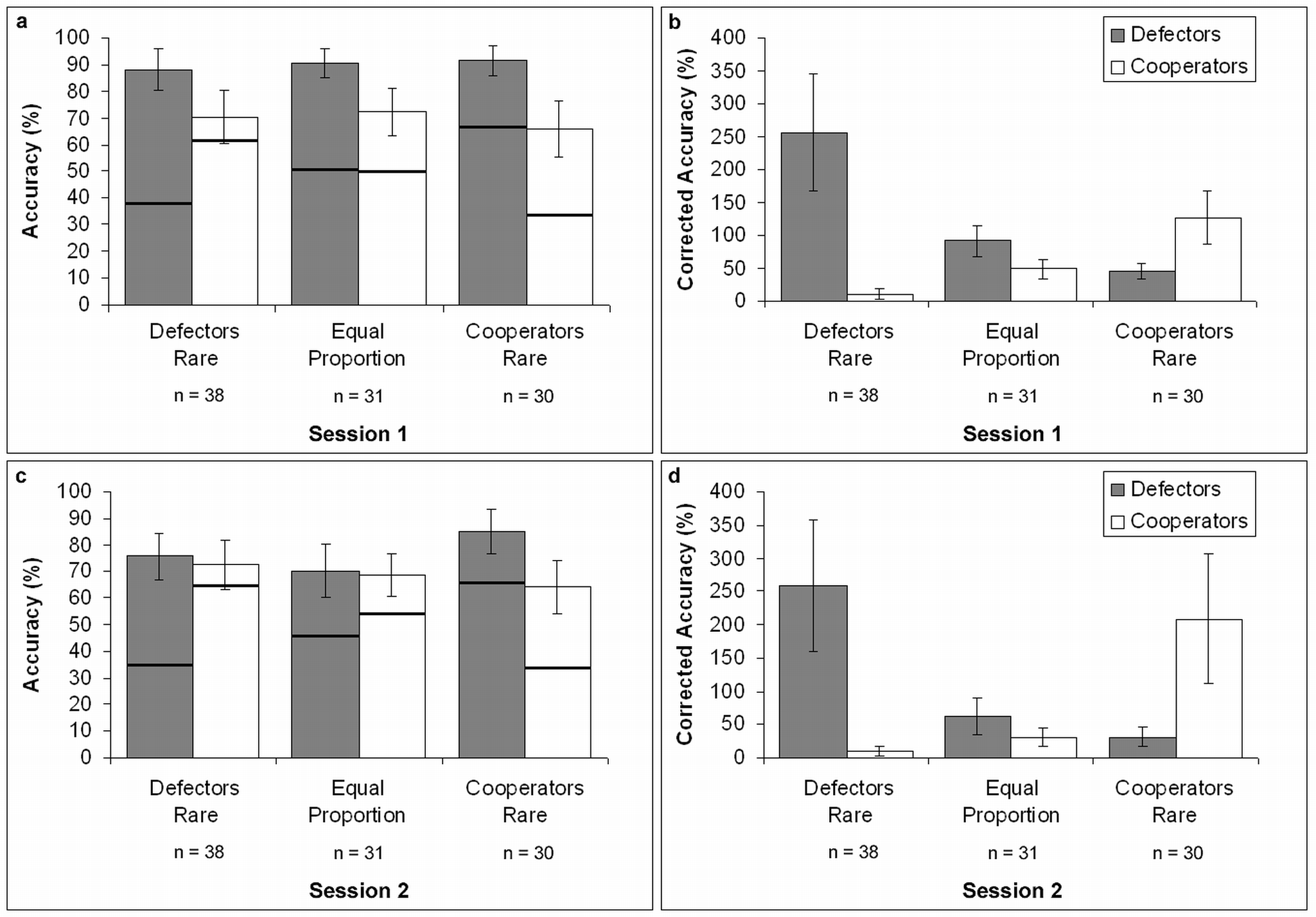

Supplement: Figure S1 — Accuracy rates using Barclay's method. Part (a) depicts the categorization accuracy for old partners independent of correct recognition (±95% confidence interval) in the three conditions in the first session. The solid line represents the chance levels based on the perceived proportion of partner types among old and new partners. In part (b), we present the relative differences between accuracy rates and chance levels using [(accuracy rate – chance level)/chance level] averaged across participants. The lower parts (c and d) show the respective results from the second session after 1 week. In the cooperators-rare condition, we averaged across n = 30 for defectors and n = 29 for cooperators in each session. (TIF) [file pone.0018945.s004.tif]
