# Supplementary material for: The Good, the Bad, and the Rare: Memory for Partners in Social Interactions
Source: PLoS One. 2011 Apr 29;6(4):e18945. doi: 10.1371/journal.pone.0018945 (PMC3084729; doi:10.1371/journal.pone.0018945)
Supplement: Table S1 — Accuracy rates for recognition, categorization independent of correct recognition, and categorization in conjunction with correct recognition (with 95% confidence intervals) from different studies investigating partner-type memory. Note. We only report partner-memory studies that provided raw values in the paper. * We calculated the 95% confidence interval from the standard deviations given. † SIM = Source Identification Measure. ‡ The values give the range of results for studies distinguishing several conditions or separating participants by gender. (DOC) [file pone.0018945.s005.doc]

Table S1

*Accur*acy rates for recognition, categorization independent of correct recognition, and categorization in conjunction with correct recognition (with 95% confidence intervals) from different studies investigating partner-type memory.

| Study | Condition | Partner Type | Recognition | Categorization independent of correct recognition | Categorization in conjunction with correct recognition (SIM†) |
| --- | --- | --- | --- | --- | --- |
| Retention interval: 0-10 min | | | | | |
| Volstorf, Rieskamp & Stevens | Defectors Rare | Defectors | 97.4 ± 3.1 | 88.2 ± 7.8 | 86.2 ± 8.8 |
|  |  | Cooperators | 99.0 ± 0.7 | 70.3 ± 9.8 | 69.7 ± 9.9 |
|  | Equal Proportion | Defectors | 99.7 ± 0.6 | 90.3 ± 5.6 | 90.0 ± 5.5 |
|  |  | Cooperators | 100.0 | 72.2 ± 8.9 | 72.2 ± 8.9 |
|  | Cooperators Rare | Defectors | 98.8 ± 1.2 | 91.5 ± 5.6 | 90.9 ± 5.7 |
|  |  | Cooperators | 98.3 ± 2.3 | 65.8 ± 10.6 | 65.0 ± 10.4 |
| Barclay (2008; personal communication) | Defectors Rare | Defectors | 81.9 ± 7.4 | 38.7 ± 9.0 | 32.5 ± 8.8 |
|  |  | Cooperators | 73.9 ± 5.7 | 80.9 ± 5.2 | 62.0 ± 6.9 |
|  | Equal Proportion | Defectors | 70.3 ± 10.5 | 58.5 ± 9.0 | 44.3 ± 10.2 |
|  |  | Cooperators | 75.7 ± 8.0 | 63.0 ± 7.6 | 51.8 ± 8.8 |
|  | Cooperators Rare | Defectors | 75.8 ± 7.3 | 85.3 ± 4.1 | 64.8 ± 6.2 |
|  |  | Cooperators | 85.0 ± 4.9 | 40.0 ± 12.9 | 35.6 ± 12.1 |
| Chiappe et al. (2004) | Equal Proportion | Defectors | 73.1 ± 0.4* | - | 42.5 ± 0.5* |
|  |  | Cooperators | 68.8 ± 0.4* | - | 35.6 ± 0.4* |
| Farrelly & Turnbull (2009) | Equal Proportion | Defectors | 47.5-57.5 ± 0.2*‡ | - | 22.5-35.0 ± 0.2*‡ |
|  |  | Cooperators | 42.5-47.5 ± 0.2*‡ | - | 25.0-27.5 ± 0.2*‡ |
| Retention interval: 1 week | | | | | |
| Volstorf, Rieskamp & Stevens | Defectors Rare | Defectors | 98.7 ± 1.8 | 75.7 ± 8.8 | 75.0 ± 9.1 |
|  |  | Cooperators | 98.4 ± 1.0 | 72.5 ± 9.4 | 71.5 ± 9.2 |
|  | Equal Proportion | Defectors | 99.0 ± 1.1 | 70.0 ± 10.1 | 69.0 ± 10.1 |
|  |  | Cooperators | 99.0 ± 1.1 | 68.7 ± 8.1 | 68.1 ± 8.1 |
|  | Cooperators Rare | Defectors | 95.4 ± 3.0 | 85.0 ± 8.4 | 82.5 ± 8.7 |
|  |  | Cooperators | 99.2 ± 1.6 | 64.2 ± 9.9 | 63.3 ± 9.9 |
| Mealey et al. (1996) | Equal Proportion | Defectors | 43.3-77.5‡ | - | - |
|  |  | Cooperators | 40.0-46.7‡ | - | - |
| Oda (1997) | Equal Proportion | Defectors | 46.7-50.0 ± 0.4-0.5*‡ | - | - |
|  |  | Cooperators | 34.4-51.1 ± 0.4-0.5*‡ | - | - |

*Note.* We only report partner-memory studies that provided raw values in the paper. * We calculated the 95% confidence interval from the standard deviations given. † SIM = Source Identification Measure. ‡ The values give the range of results for studies distinguishing several conditions or separating participants by gender.
